# The Value of Local Anæsthetics

**Published:** 1900-07

**Authors:** 


### Domestic Correspondence.

### THE VALUE OF LOCAL ANÆSTHETICS.

To the Editor:

Sir,—I am engaged in an investigation of the value of local anæsthetics, and members of the dental profession can materially aid me by answering the questions appended. There is such a marked diversity of opinion as to the propriety of applying local anæsthetics about the mouth, that any attempt to arrive at a statistical conclusion should meet not alone with favor, but with the hearty co-operation of the profession at large. Few dental institutions of learning advocate or teach the use of local anæsthetics, and still, as each graduate enters upon his professional career, he is confronted by the important question, Is one justified in using local anæsthesia to alleviate the pain in dento-surgical operations?

Some men are firm advocates of the use of local anæsthetics, others strongly condemn them. If one man gets good results with the refrigerating spray or the cocaine-charged syringe and the other does not, investigation may show that they are at variance in their methods of application.

When concerted opinion is absent in the profession, who is to decide in an individual case whether the post-operative swelling or sloughing is due to the traumatism, infection of the part, or the toxic effect of the anæsthetic?

Many important points of this nature should be brought out in this investigation, and I kindly request that answer in full be made to the questions mentioned. Space is provided upon the enclosed question slip for the report of any interesting cases bearing upon this subject.

Due credit will be given for all information.

MORRIS I. SCHAMBERG, D.D.S., M.D.,
*Late Acting Assistant Surgeon, U. S. A.; Quiz-Master in Oral Surgery, Association of Quiz-Masters, University of Pennsylvania; Clinical Assistant in Surgery, Polyclinic Hospital, Philadelphia.*
1636 WALNUT STREET, PHILADELPHIA, MAY 10, 1900.

QUESTIONS.

1. Do you employ local anæsthesia in your practice?
2. Kindly state what drugs or combination of drugs used for this purpose, and also your method of employing them.
3. Have you observed any untoward effects, either constitutional or local, from their use?
4. What means, if any, do you find necessary to prevent post-operative swelling and sloughing?

Remarks or reports of interesting cases.

